# Endoscopic Debulking in the Management of Osteosarcoma With a Rare Endobronchial Metastasis

**DOI:** 10.7759/cureus.103501

**Published:** 2026-02-12

**Authors:** Jeffrey S Jang, Ghita Bouzarif, David W Hsia

**Affiliations:** 1 Department of Medicine, Harbor-University of California Los Angeles Medical Center, Torrance, USA; 2 Division of Respiratory and Critical Care Physiology and Medicine, Department of Medicine, Harbor-University of California Los Angeles Medical Center, Torrance, USA; 3 Division of Respiratory and Critical Care Physiology and Medicine, Lundquist Institute for Biomedical Innovation at Harbor-UCLA Medical Center, Torrance, USA

**Keywords:** bronchoscopy, central airway tumors, endobronchial metastasis, metastatic osteosarcoma, rare osteosarcoma

## Abstract

Osteosarcoma most commonly metastasizes to the lung, which requires individualized management based on the location of the disease. Surgical resection has been the cornerstone to improving outcomes in peripheral metastatic lung disease. However, central airway obstruction is rare, resulting in limited evidence regarding the optimal management of this manifestation. We report a case of a 68-year-old man with osteosarcoma and bilateral lung metastasis. A 10 centimeter tumor on the right involved the hilum with extension to the pleura. Bronchoscopy confirmed an endobronchial mass occluding the bronchus intermedius. This central airway tumor was debulked via endoscopy and later managed medically with chemotherapy. This case describes a rare manifestation of osteosarcoma metastasis and the multidisciplinary management to control tumor burden.

## Introduction

Osteosarcoma is mainly a disease of children and young adults that can present later in life [1]. The most common site of metastasis is the lung parenchyma [2], which necessitates multidisciplinary management including medical and procedural options given that distant dissemination is responsible for the majority of osteosarcoma-related mortality. Although lung metastasis is well-known in this malignancy, metastasis to the central airway is much less described, accounting for approximately 1% of cases [3]. Symptoms of osteosarcoma with endobronchial disease include coughing, dyspnea, and hemoptysis [4,5]. Given the rarity, treatment options are less defined. Surgical resection generally improves survival rates in select patients with pulmonary metastatic involvement [6]; however, endobronchial tumor burden presents a greater challenge with limitations for definitive surgical or procedural options.

## Case presentation

A 68-year-old man with hypertension, hyperlipidemia, and osteosarcoma of the right femur with lung metastasis presented to the emergency department with hemoptysis and dyspnea. 

The patient’s oncological history was notable for osteosarcoma of the right femur requiring above the knee amputation. Pathology of surgical specimens confirmed Grade 2/Grade 3 osteosarcoma with areas of chondroid, fibrous, and osteoclast differentiation. Repeat CT chest computed tomography (CT) scan of the chest two months later showed multi-lobar nodules concerning for progression of disease. He underwent six cycles of doxorubicin and cisplatin with subsequent imaging showing stable nodules. The patient was lost to follow up after completion of chemotherapy. He presented one year later to the emergency department for cough, and CT demonstrated a large mass with involvement of the hilum extending in the lumen of the right lower lobe bronchial lumen. New bilateral pulmonary nodules were also identified. The patient was again lost to follow up until nine months later with the presentation of hemoptysis.

**Figure 1:**
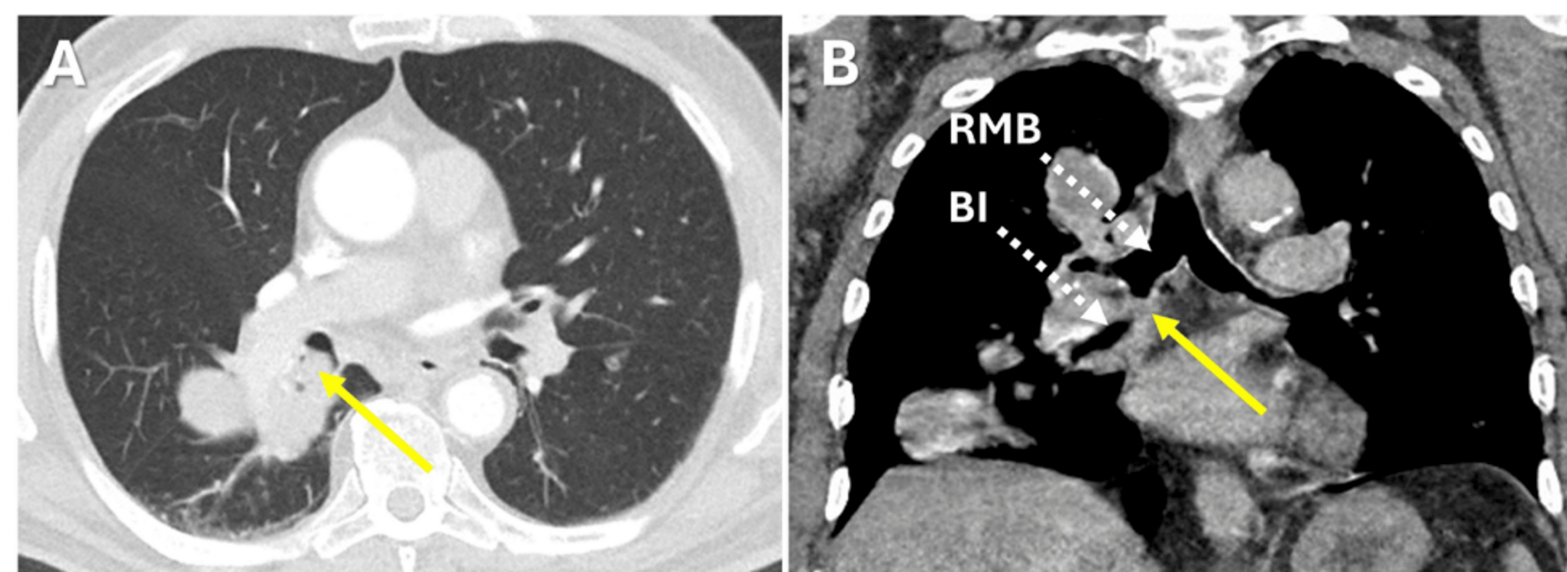
Axial (Panel A) and sagittal (Panel B) CT chest images demonstrating endobronchial metastasis (yellow arrows) causing obstruction between the right mainstem bronchus (RMB) and bronchus intermedius (BI).

In the most recent presentation to the emergency department, he endorsed non-massive hemoptysis at least five times a day for four days. His vitals signs included a respiratory rate of 18 breaths per minute and an oxygen saturation of 98% on room air. On physical examination, he appeared well-nourished. His respirations were non-labored, but he had decreased breath sounds in the right lower lung field. His right lower extremity was amputated. Laboratory results were notable for a decreased hemoglobin at 11.9 g/dL. Chest CT scan revealed multiple lesions, including a 10 centimeter obstructing right lower lobe mass involving the hilum and extending to the pleura (Figure 1). Bilateral pulmonary nodules had increased in size including a 22 millimeter nodule in the right upper lobe nodule and an 18 millimeter left lower lobe nodule.

Flexible bronchoscopy with procedural sedation revealed a large, well-circumcised endobronchial mass completely occluding the bronchus intermedius. Given the friability of the mass, endobronchial biopsies were not performed, with plans for repeat rigid bronchoscopy. Given the complexity of the case due to the location of metastasis, the patient's case was discussed as part of a multidisciplinary conference including the Pulmonology and Oncology departments. The extent of the disease including central airway involvement was determined to necessitate a pneumonectomy for surgical resection which, in addition to bilateral lung nodules, precluded him as a surgical candidate for metastasectomy. Since the patient had a previous history of metastatic disease, outside records were obtained that confirmed a history of osteosarcoma metastasis to the lungs. The multidisciplinary discussion included deciding the appropriate timing of chemotherapy, and whether it was optimal to do prior to or after bronchoscopic debulking. Eventually, the decision was made to debulk the occlusive endobronchial tumor first and follow with chemotherapy. 

**Figure 2:**
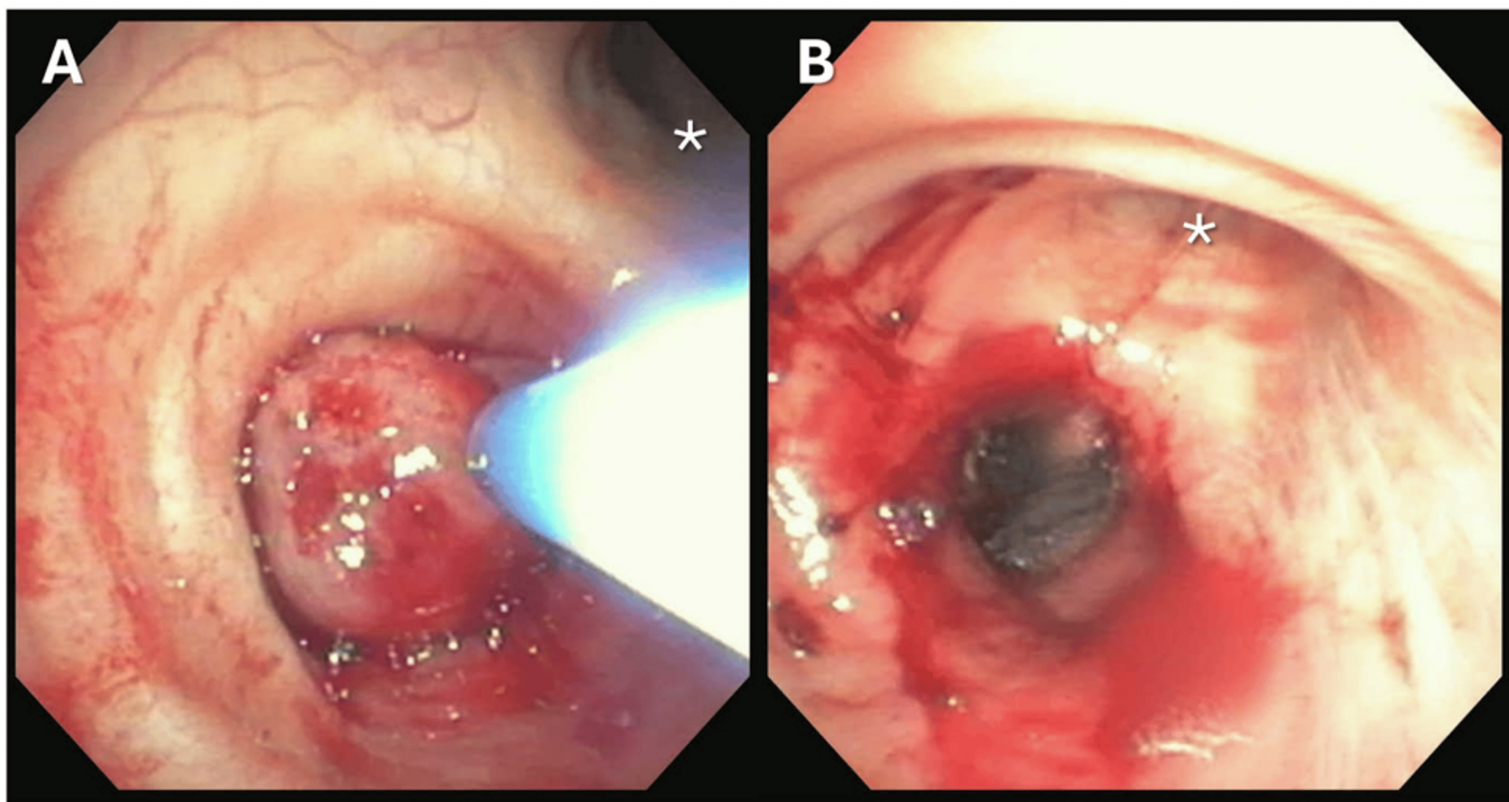
Figure 2. Bronchoscopy view (BF-1T180, Olympus America, Inc.) from the right mainstem bronchus demonstrating a large exophytic endobronchial mass completely occluding the bronchus intermedius with electrocautery snare prior (Panel A) and after resection and ablation (Panel B). For reference, the right upper lobe is indicated by an asterisk.

Rigid bronchoscopy was performed with general anesthesia demonstrating a broad-based tumor in the bronchus intermedius. Electrocautery snare resection, tumor ablation with cautery probe and argon plasma coagulation (APC), and tumor debulking with cryoprobe and forceps resulted in successful recannulization of more than 50% of the bronchus intermedius lumen (Figure 2). 

The patient tolerated the procedure well and pathology confirmed CD99-positive metastatic sarcoma consistent with the original disease. The patient continued therapy with ifosfamide and etoposide. Repeat CT scans showed interval reduction in bilateral metastatic nodule size. The right lower lobe mass was also reduced but maintained involvement in the hilum extending to the pleura. Table 1 outlines the timeline of the patient's presentation and treatment. 

**Table 1:** Timeline of key clinical events

Timeline	Event	Teams
Time 0	Presentation with right leg soft tissue mass requiring above-the-knee amputation confirming diagnosis of osteosarcoma	Emergency Medicine, Orthopedics, Pathology
3 months	Initiated treatment with doxorubicin and cisplatin. CT evidence of bilateral pulmonary nodules.	Oncology
7 months	Completed chemotherapy. Subsequently lost to follow-up.	Oncology, Orthopedics
44 months	Emergency department visit for cough. CT showing right lower lobar mass with extension to bronchial lumen. Lost to follow-up.	Emergency Medicine
53 months	Emergency department visit for hemoptysis.	Emergency Medicine, Interventional Pulmonology
59 months	Multidisciplinary conference. Discussed plan for bronchoscopy debulking and chemotherapy.	Interventional Pulmonology, Oncology, Cardiothoracic Surgery, Pathology
60 months	Bronchoscopy with debulking.	Interventional Pulmonology
60 months	Cycle 1 etoposide and ifosfamide.	Oncology
64 months	CT with evidence suggestive of partial response.	Oncology

## Discussion

Osteosarcoma predominantly affects children and young adults; interestingly, incidence rises after age 60 [1]. Approximately 15-20% of patients present with metastatic disease, most commonly to the lung parenchyma [2]. Endobronchial metastases from osteosarcoma is exceedingly rare and described in only a handful of cases [4,5,7-10]. These cases were not always treated in the same manner, and each provides unique insight to managing this uncommon metastasis location. In a review of CT findings from 127 patients, osteosarcoma presented with endobronchial lesions in less than 1% of cases [10]. 

First-line therapy for osteosarcoma includes cisplatin and doxorubicin, which is a regimen that has been used for decades. High-dose methotrexate is now included in current guidelines with ifosfamide demonstrating efficacy as a second-line therapy [2]. 

Metastatic lung disease management hinges on surgical resection when feasible. Metastasectomy may improve survival for lung metastases, including improvements in the five-year survival to 28-41% with radical pulmonary metastasectomy in adults [3,11]. There are a variety of surgical approaches, however the role of surgery for central airway tumors has been less described. In our patient’s case, the proximity to the hilum and involvement of the bronchus intermedius would necessitate pneumonectomy as a resection approach. Pneumonectomy remains limited to select cases due to the high morbidity risk [12]. In a large retrospective study of pulmonary metastasis, only 1% were observed to have undergone pneumonectomy [13] but is not an option in bilateral disease [7].

A recent retrospective study regarding metastasectomy of osteosarcoma metastasis showed that resection of central airway disease resulted in a five-year overall survival rate of 17.6%. Notably, the study’s criteria for central airway involvement included tumor abutting a first or second-degree blood vessel or bronchus [14]. Our case represents one of a limited number of osteosarcoma cases with confirmed endobronchial involvement with tumor occlusion of the airway described in the medical literature. The paucity of evidence on endobronchial disease of osteosarcoma presents a dilemma in selecting the appropriate procedural option. Our patient’s bilateral tumor burden excluded the option for pneumonectomy. At present, pneumonectomy lacks consensus as the primary management of central disease, and examination of other procedural options is warranted. For patients not considered surgical candidates, bronchoscopy provides a means of palliating the complications resulting from an obstructing endobronchial mass.

Bronchoscopy debulking is an option to relieve obstruction from central airway tumors, though it does not provide a method of complete resection and is therefore used as palliation and not curative intent. Given the rarity of endobronchial osteosarcoma, no studies specifically focus on outcomes of bronchoscopy with central airway obstruction from this disease, and recommendations for bronchoscopic management are extrapolated from bronchoscopic interventions for lung cancer and other pulmonary metastases. Mechanical debulking via bronchoscopy has shown to increase overall survival, especially when combined with systemic therapy. It is also a procedure used for palliation of symptoms of central airway obstruction in patients who are not surgical candidates [15-17]. Patient selection is crucial as symptom benefits primarily result from recanalization of airways obstructing otherwise functional distal lung parenchyma or the temporary ablation of tumor bleeding resulting in hemoptysis. Additional benefits include the reduction of post-obstructive complications such as recurrent infections or difficulty with mucous clearance. In this case, palliative endobronchial tumor ablation and debulking was used as the patient had radiographic evidence of functional lung distal to the obstruction.

Interventional bronchoscopic techniques to debulk tumors include the use of flexible and rigid bronchoscopes. Endobronchial tumor ablation and debulking of osteosarcomas have been performed through a variety of techniques cryotherapy, electrocautery, and argon plasma coagulation [5,8] While each case may provide different rationale for choice of debulking method, the experience and preference of the operator normally guides the optimal option. No randomized trials have been conducted comparing outcomes based on techniques or equipment utilized for central airway tumors [16]. Regardless of the option, debulking central airway tumors via bronchoscopy has been shown to alleviate symptoms and improve quality of life [15-16]. A guideline statement from the American College of Chest Physicians gives a Conditional Recommendation for the use of therapeutic bronchoscopy as an adjunct to systemic and radiation therapy [18].

As there is no single defined treatment for management of osteosarcoma with endobronchial disease, different cases have addressed the situation differently. Previous teams have utilized bronchoscopic resection, similar to our case [5,8]. One case achieved complete response after therapy with intraluminal radiotherapy [10]. One report described surgical management via pneumonectomy in two different patients [7]. Each case represents a unique presentation with ranges in age and disease burden. Accounting for patient specific features is crucial in managing this challenging metastatic presentation of osteosarcoma. Table 2 outlines reported cases of osteosarcoma with endobronchial metastasis.

**Table 2:** Reported cases of osteosarcoma with endobronchial metastasis

Author (year)	Age	Location of metastasis	Osteosarcoma features	Management (bronchoscopy, surgery, and other)	Outcome
Doherty et al. (1996) [19]	30	Right bronchus intermedius and left main bronchus	Chondroblastic	Rigid bronchoscopy with forceps	Symptomatic improvement, but later died from extra-pulmonary disease burden.
Syriac et al. (2019) [8]	36	Left lower lobe	Osteosarcoma, unspecified	Bronchoscopy cryotherapy and argon plasma coagulation	Resolution of symptoms and mild reduction in pulmonary metastasis.
Kumar et al.(2023) [5]	20	Right upper lobe	High-grade pleomorphic	Bronchoscopy cryotherapy, balloon dilation, argon plasma coagulation	Tolerated procedure with improvement of symptoms.
Jang et al. (2026)	68	Right mainstem bronchus	High grade with chondroid, fibrous and osteoclast differentiation	Bronchoscopy cryoprobe and forceps debulking, electrocautery snare resection, and argon plasma coagulation	Tolerated procedure with 50% recannulation. Stable chest imaging at 3 months and without symptoms.
Akiba et al. (1994) [20]	73	Left basal bronchus	Spindle-shaped cells and multinucleated osteoclast-like giant cells	Lobectomy	Symptomatic improvement, progression of pulmonary metastasis.
Attar et al. (2024) [7]	29	Right main bronchus	Spindle-cell proliferation with osteoid formation	Pneumonectomy	Concern for relapse at bronchial stump.
Attar et al. (2024) [7]	45	Left upper lobe	Atypical spindle-cell proliferation	Pneumonectomy	Disease free at 46 months.
Mogulkoc et al.(1999) [10]	21	Right main bronchus	Osteosarcoma, unspecified	Single treatment of intraluminal radiotherapy	Repeat bronchoscopy without endobronchial tumor. Patient died 86 days later.
Ludwigsen (1977) [9]	76	Left main bronchus	Polymorphic osteosarcoma	Died during biopsy	Death from hemorrhage during biopsy.
Kayal et al. (2013) [4]	29	Right main bronchus	Osteosarcoma, unspecified	Died prior to treatment	Death from massive hemoptysis before treatment.

## Conclusions

We describe a patient with osteosarcoma and rare endobronchial metastasis. Tumor involvement of the central airway was managed with endoscopic debulking. The patient tolerated subsequent chemotherapy with ifosfamide and etoposide with a treatment goal of progression free survival. At an outpatient pulmonology appointment three months after the endoscopy, he denied recurrence of hemoptysis. He continued to undergo surveillance CT scans. 

The challenging location of the disease prompted a multidisciplinary approach outlining the roles for surgery, chemotherapy, and interventional pulmonology. This case demonstrates the use of endoscopy to alleviate obstruction of the central airway prior to systemic oncological therapy. It highlights the importance of multiple specialties to determine the course of action best suited for challenging cases. Endobronchial metastasis in osteosarcoma requires a patient-centered approach with consideration for medical and procedural management.
